# Increased expression of integrin-linked kinase is associated with shorter survival in non-small cell lung cancer

**DOI:** 10.1186/1471-2407-5-1

**Published:** 2005-01-05

**Authors:** Iwao Takanami

**Affiliations:** 1 First Department of Surgery, Teikyo University School of Medicine, Tokyo, Japan

## Abstract

**Background:**

Integrin-linked kinase (ILK) promotes tumor growth and invasion. Increased ILK expression is correlated with progression of several tumor types, but the expression of ILK has not been investigated in patients with non-small cell lung cancers (NSCLCs).

**Methods:**

We investigated ILK expression in patients with NSCLC by means of immunohistochemistry.

**Results:**

ILK expression was significantly associated with tumor grade, T status, lymph node metastasis and stage. (p = 0.0169 for tumor grade; p = 0.0006 for T status; p = 0.0002 for lymph node metastasis; p < 0.0001 for stage). The 5-year survival rates for patients with strong and weak or no ILK expression levels were 20% and 59%, respectively: the difference was statistically significant (p < 0.0001). A multivariate analysis of survival revealed that ILK expression, T status, N status and vascular invasion were statistically significant prognostic factors (p = 0.0218 for ILK; p = 0.0046 for T status; p < 0.0001 for N status; p < 0.0001 for vascular invasion).

**Conclusions:**

Our study demonstrates that increased expression of ILK is a poor prognostic factor in patients with NSCLC.

## Background

Interaction of cells with the extracellular matrix regulates many physiological and pathological processes. These interactions are mediated by a large family of cell surface receptors known as integrins, which recognize several extracellular matrix proteins, including fibronectin, collagens, and vitronectin [1]. Integrins act as the bridge between extracellular matrix components and the cytoskeleton and other proteins, regulating cell survival, proliferation, differentiation, and migration [1]. Integrins are important mediators of tumor invasion and metastasis through interaction with extracellular matrix. All integrins are heterodimers composed of one copy each of two subunits, α and β. Many studies examined the association between integrins and clinicalpathology or prognosis in lung cancer. Reduced integrin α3β1 expression was reported to be a factor of poor prognosis in pulmonary adenocarcinoma [2]. Increased expression of integrin β1 was reported to be a poor prognosis in small cell lung cancer [3]. Integrin α5β1 was reported to be associated with lymph node metastasis of non-small cell lung cancer (NSCLC) [4], or to be a prognostic factor in node-negative NSCLC [5].

Integrin-linked kinase (ILK) interacts with cytoplasmic domain of both β1 and β3 integrins and is activated by cell- extracellular matrix interactions [6]. Overexpression of ILK in epithelial cells results in anchorage-independent cell growth with increased cell cycle progression [7]. Furthermore, increased ILK expression is correlated with progression of several tumor types, including prostate [8], gastric [9], and colon carcinomas [10]. However, to our knowledge, the expression of ILK has not been investigated in patients with NSCLC. Thus, we investigated ILK expression in series of 134 cases of curatively resected NSCLC by means of immunohistochemical assays to evaluate its clinical significance.

## Methods

### Patients

A total of 134 patients (88 men and 46 women; mean age, 65 years; age range, 28 to 80 years) with NSCLC were studied (consecutive cases). All patients in this study had undergone curative tumor resection in our department between 1995 and 1998. Patients who died within 30 days after surgery and those who underwent exploratory thoracotomy were excluded from the study. Patients with a past history of another type of cancer were also excluded. With regard to histological type, 75 were adenocarcinomas, 54 were squamous cell carcinomas and 5 were large cell lung carcinomas. The lesions of these 134 patients were staged on both operative and pathologic findings according to the UICC TNM classification [11] (1997). There were 36 patients (27%) with stage Ia, 37 (28%) with stage Ib, 3 (2%) with stage IIa, 18 (13%) with stage IIb, and 40 (30%) with stage IIIa. None of the study subjects received pre- or postoperative chemotherapy and median follow-up of the patients was 75.3 months (range 60–96 months) and their outcomes were known.

### Immunohistochemical staining

Resected tissue specimens were fixed in formalin, embedded in paraffin, and cut into 3-μm serial sections. The sections were subjected to hematoxylin-eosin staining and immunohistochemical analysis with ILK. A mouse anti-ILK monoclonal antibody (Santa Cruz Biotechnology, CA, USA) was used, and immunohistochemical staining was based on the avidin-biotin-peroxidase complex method and was performed with a Vestastatin kit (Vector, Burlingame, CA). Negative control sections were treated using nonspecific IgG in the primary antibody.

### Determination of expression of ILK

Immunoreactivity was graded as from - to +++, according to the number of cells stained. Grades was defined as follow as: -, no positive cells; ±, less than 5% of tumor cells showed immunoreactivity; +, 5–50% of tumor cells showed immunoreactivity; ++, 50–80% of tumor cells showed immunoreactivity; +++, over 80% of tumor cells showed immunoreactivity. Grades ++ and +++ were regarded as strong expression, and grades ± and + were regarded as weak expression. The results of immunohistochemical staining were evaluated independently by three observers with no prior knowledge of patients' clinical data. The evaluation was suitable for 96% of the samples. The other specimens (4%) were re-evaluated independently, then classified according to the classification given most frequently by the observers. In this study, we compared the group of tumors of strong ILK expression with the group of tumors of weak or no ILK expression.

### Statistical analysis

The association between ILK expression and clinical data was statistically evaluated by using Mann-Whitney U test or the chi-square test. Survival curves were calculated by the Kaplan-Meier method and were compared using the log-rank test. The correlation of variables with survival was analyzed by multivariate analysis using a Cox proportional hazards model. All statistical analyses were performed using the StatView software package (Abaracus Concepts, Berkeley CA). A P value of <0.05 was considered statistically significant.

## Results

### Expression of ILK and clinicopathologic parameters

**Figure 1:**
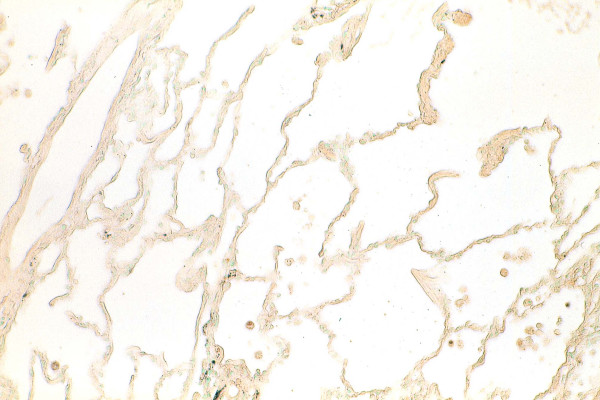
Immunohistochemical studies of ILK in non-neoplastic pulmonary tissue and in NSCLC tissue. a non-neoplastic pulmonary tissue: ILK was not detected in epithelial cells, while ILK expression was found in many stromal cells.

**Figure 2:**
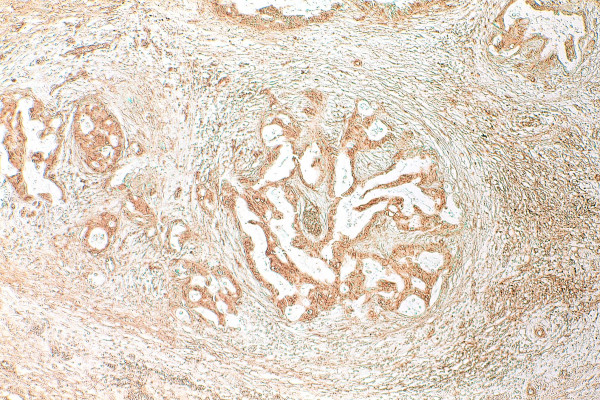
well differentiated adenocarcinoma: ILK protein was localized in both cytoplasms and nuclei. (*Magnification*, ×40 (Figure 1), ×16 (Figure 2), and ×40 (Figure 3)).

**Figure 3:**
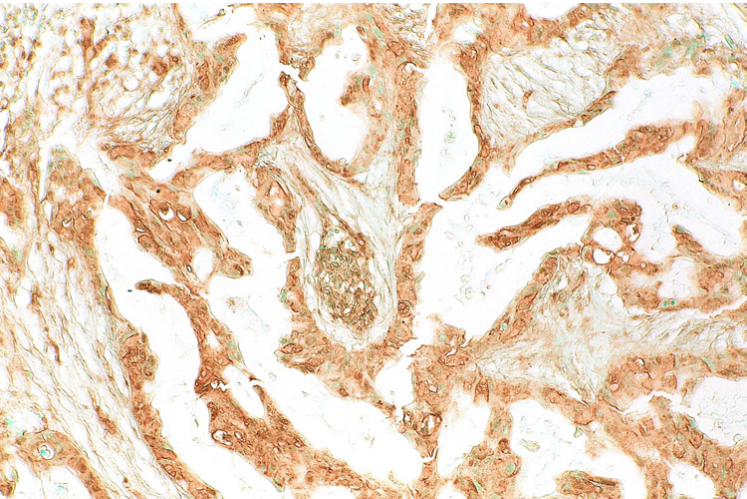
well differentiated adenocarcinoma: ILK protein was localized in both cytoplasms and nuclei. (*Magnification*, ×40 (Figure 1), ×16 (Figure 2), and ×40 (Figure 3)).

In non-neoplastic lung tissue, ILK was not detected in epithelial cells, while ILK expression was found in stromal cells including fibroblasts and infiltrating lymphocytes (Figure 1). In the cancer cells of many patients, ILK expression was detected in both cytoplasm and nuclei (Figure 2, and 3). Of the 134 patients, 6 (4%) were classified as -, 34 (25%) as ±, 53 (40%) as +, 25 (19%) as ++, and 16 (12%) as +++. Patients with ILK ++ and +++ were placed together in a strong ILK group and were compared with weak or no ILK group. Tumor cells expressed ILK protein in most NSCLC cases, and strong expression was detected 31% (41 of 134) of the cases. Stromal cells in cancer lesion were also positive to ILK, and rate of positive cells and intensity of the staining were not different from those of stromal cells in non-neoplastic lesion. As shown in Table 1, there were no significant differences between ILK expression status and clinical factors of age, gender, histology, lymphatic invasion and vascular invasion. However, the expression of ILK protein was significantly associated with tumor grade, T status, lymph node metastasis and stage (p = 0.0169 for tumor grade; p = 0.0006 for T status; p = 0.0002 for lymph node metastasis; p < 0.0001 for stage).

### Relationship between ILK expression and overall survival

**Figure 4:**
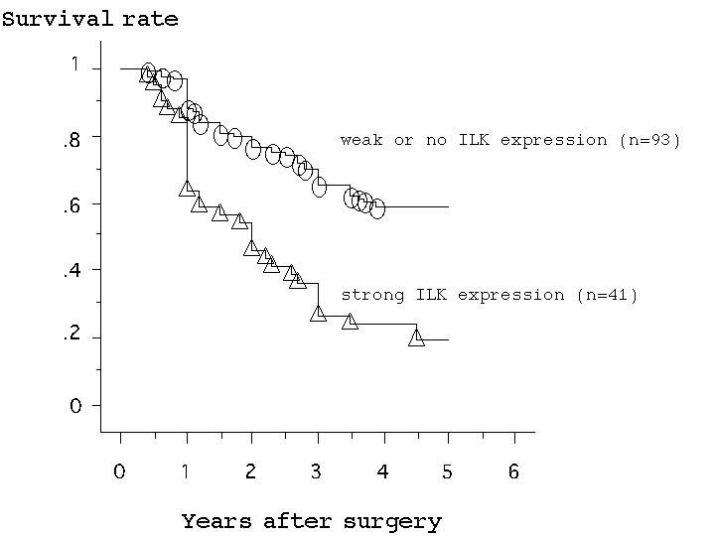
Overall survival curves for patients classified according to expression of ILK. Five-year survival rates were 20% and 59% for patients in the strong ILK and weak or no ILK groups, respectively. A log-rank test revealed a significant difference between the two groups (p < 0.001).

The 5-year survival rates for the groups with strong, and weak or no for ILK expression in their tumors were 20% and 59% respectively: the difference was statistically significant (p < 0.0001) (Figure 4). The univariate survival analysis revealed that tumor grade, T status, N status, stage, lymphatic invasion, vascular invasion and ILK expression all were statistically significant prognostic factors (p = 0.0003 for tumor grade; p < 0.0001 for T status; p < 0.0001 for N status; p < 0.0001 for stage; p < 0.0001 for lymphatic invasion; p < 0.0001 for vascular invasion; p < 0.0001 for ILk expression) (Table 2). However, age, gender, and histology were not significant factors. The multivariate survival analysis revealed that T status, N status, vascular invasion and ILK expression were statistically significant prognostic factors (p = 0.0046 for T status; p < 0.0001 for N status; p < 0.0001 for vascular invasion; p = 0.0218 for ILK expression) (Table 3).

## Discussion

Recent studies have indicated that ILK facilitated the phosphorylation of AKT, which is required for AKT activation [12]. Activation of AKT upregulates vascular endothelial growth factor expression, and AKT is known to induce angiogenesis and suppress apoptosis [12]. By regulating, the activity of AKT as well as glycogen synthase kinase 3, ILK facilitates the assembly and activity of the β-catenin/LEF-1 transcription complex, and suppresses expression of the invasion suppressor E-cadherin [13,14]. Overexpression of ILK in epithelial cells results in anchorage-independent cell growth with increased cell cycle progression, and constitutive up-regulation of cyclin D and cyclin A expression [7]. Inhibition of ILK elicits cell cycle arrest and induces apoptosis [15]. Overexpression of ILK in epithelial cells induces tumorigenicity in nude mice, indicating that ILK can act as a proto-oncogene [16]. ILK is associated with a highly invasive phenotype of certain tumors [7].

In the current study, we detected ILK expression using immunohistochemistry on tumors from patients with NSCLC. ILK was strongly expressed in 31% of tumor samples, whereas there was no ILK expression in noncancerous pulmonary tissue samples from the same patients, except for fibroblasts and infiltrating lymphocytes. These finding suggest that ILK expression may serve as a useful marker of NSCLC. ILK is very low in healthy cells. In cancer cells, however, ILK activity is often increased, possibly as a result of a malfunctioning of upstream components in the integrin and growth factor signaling pathways. We found a significant correlation between strong expression and advanced T status, N status and stage. No correlation was found age, gender or histology. These results suggest that ILK expression is correlated with tumor progression in NSCLC. Cases with strong ILK expression were reported to be significantly more frequent in advanced gastric carcinoma [9]and advanced melanoma [17]. ILK-mediated pathway that may enhance tumor progression is its regulation on MMP expression [18]. During tumor progression, MMPs facilitate the pathological processes of tumor invasion, angiogenesis, and metastasis by breaking down the extracellular matrix. The overexpression of ILK has been shown to be result of increased MMP-9 expression [18]. We also demonstrated that the expression of ILK correlated with tumor grade. Recent studies have also linked ILK expression to tumor grade of prostate [7], gastric [9] or ovarian carcinomas [19]. The overall prognosis of patients with strong ILK expression was significantly poorer than that of patients with weak or no expression. For stage I or stage II/IIIa patients, prognosis was poorer for those with strong ILK expression than for those with weak or no ILK expression, although the differences were not significant (data not shown). Strong expression of ILK protein was reported to be significantly associated with presence of nodal metastasis [9,17]. The univariate survival analysis revealed ILK expression was a significant prognostic factor as well as T status, N status, stage, lymphatic invasion and vascular invasion. The status of ILK expression might be dependent on the status of lymph node metastasis or other variables. So the multivariate analysis for survival was performed. In the current study, the multivariate analysis revealed that ILK expression was picked up for its independent level of prognostic significance. Our results also showed that the tumors with a strong expression of ILK were associated with an increased recurrence, which suggests that patients with strong ILK expression may be prone to metastasis, or may already have occult systemic diseases. ILK expression level plays one of the key roles in the biology of NSCLC and defines a more aggressive tumor phenotype of NSCLC. Preoperative adjuvant therapy in NSCLC is designed to improve survival and reduce local recurrence. Recent reports have shown that preoperative adjuvant therapy has led to an increase in overall survival of NSCLC patients [20,21]. It is possible that this preoperative adjuvant treatment modality may be important for patients whose tumors have strong ILK expression. In the future, small molecule antagonists of ILK may be used to interfere with recurrence in tumor patients.

## Conclusion

Our study demonstrates that ILK expression is a poor prognostic factor in patients with NSCLC. Thus, the utility of the expression of ILK could open up a new window for the molecular marker and the treatment of NSCLC.

## Competing interests

The author(s) declare that they have no competing interests.

## Pre-publication history

The pre-publication history for this paper can be accessed here:



## Figures and Tables

**Table 1:** Clinicopathologic characteristics of patients with NSCLC classified according to ILK

Characteristic	Expression of ILK		P-value
		
	weak or none (n = 93)	strong (n = 41)	
Age			
MEAN ± SD	64.6 ± 11.0	64.5 ± 9.3	0.3412

Gender			
Male	57	31	
Female	36	10	0.1077

Histology			
Adenocarcinoma	54	21	
Squamous cell ca.	37	17	
Large cell ca.	2	3	0.4771

Tumor grade			
Well differentiated	38	9	
Mode differentiated	37	15	
Poorly differentiated	18	17	0.0169

T status			
T1	34	6	
T2	49	20	
T3	10	15	0.0006

Nodal status			
N0	69	18	
N1	7	1	
N2	17	22	0.0002

Stage			
Ia/Ib	62	11	
IIa/IIb	14	7	
IIIa	17	23	<0.0001

Lymphatic invasion			
Negative	42	15	
Positive	51	26	0.3548

Vascular invasion			
Negative	56	19	
Positive	37	22	0.1360

**Table 2:** Univariate Analysis of Variables that Affected the Overall Survival Rate Determined by Cox Proportional Hazards Model

Variable	Relative risk	95%CI	p-value
Age (<65 yrs/≦65 yrs)	1.070	0.699–1.710	0.080
Gender (male/female)	1.537	0.866–2.383	0.1605
Histology (SCC/others)	1.174	0.688–2.005	0.5560
Tumor grade (mode., poor/well)	1.802	1.130–2.478	0.0003
T status (T2, T3/T1)	2.669	1.878–3.794	<0.0001
N status (N1, N2/N0)	5.911	3.600–9.705	<0.0001
Stage (stage II, III/Stage I)	8.143	4.851–12.255	<0.0001
Lymphatic invasion (positie/negative)	3.634	2.074–6.367	<0.0001
Vascular invasion (positive/negative)	3.198	1.974–5.181	<0.0001
ILK (strong/weak or no)	2.926	1.827–4.686	<0.0001

**Table 3:** Risk Factors that Affected the Overall Survival Rate Determined by the Cox Proportional Hazards model

Variable	Relative risk	95%CI	p-value
Tumor grade (mod., poor/wel)	1.238	0.858–1.786	0.2543
T status (T2, T3/T1)	1.845	1.208–2.818	0.0046
N status (N1, N2/N0)	3.897	2.241–6.775	<0.0001
Vascular invasion (positive/negative)	2,981	1.733–5.127	<0.0001
ILK (strong/weak or no)	1.844	1.093–3.112	0.0218
